# The effects of hypertonic fluid administration on the gene expression of inflammatory mediators in circulating leucocytes in patients with septic shock: a preliminary study

**DOI:** 10.1186/2110-5820-1-44

**Published:** 2011-11-01

**Authors:** Frank MP van Haren, James Sleigh, Ray Cursons, Mary La Pine, Peter Pickkers, Johannes G van der Hoeven

**Affiliations:** 1 Intensive Care Department, The Canberra Hospital, Canberra, Australia; 2 Intensive Care Department, Waikato Hospital, Hamilton, New Zealand; 3 Molecular Genetics Laboratory, University of Waikato, New Zealand; 4 Intensive Care Department, Radboud University Nijmegen Medical Centre, Nijmegen, The Netherlands

## Abstract

**Objective:**

This study was designed to investigate the effect of hypertonic fluid administration on inflammatory mediator gene expression in patients with septic shock.

**Design and setting:**

Prospective, randomized, controlled, double-blind clinical study in a 15-bed mixed intensive care unit in a tertiary referral teaching hospital.

**Interventions:**

Twenty-four patients, who met standard criteria for septic shock, were randomized to receive a bolus of hypertonic fluid (HT, 250 ml 6% HES/7.2% NaCl) or isotonic fluid (IT, 500 ml 6% HES/0.9% NaCl) administered over 15 minutes. Randomization and study fluid administration was within 24 hours of ICU admission for all patients. This trial is registered with ANZCTR.org.au as ACTRN12607000259448.

**Results:**

Blood samples were taken immediately before and 4, 8, 12, and 24 hours after fluid administration. Real-time reverse transcriptase polymerase chain reaction (RT rtPCR) was used to quantify mRNA expression of different inflammatory mediators in peripheral leukocytes. In the HT group, compared with the IT group, levels of gene expression of MMP9 and L-selectin were significantly suppressed (*p *= 0.0002 and *p *= 0.007, respectively), and CD11b gene expression tended to be elevated (*p *= NS). No differences were found in the other mediators examined.

**Conclusions:**

In septic shock patients, hypertonic fluid administration compared with isotonic fluid may modulate expression of genes that are implicated in leukocyte-endothelial interaction and capillary leakage.

The study was performed at the Intensive Care Department, Waikato Hospital, and at the Molecular Genetics Laboratory, University of Waikato, Hamilton, New Zealand.

**Trial registration:**

Australia and New Zealand Clinical Trials Register (ANZCTR): ACTRN12607000259448

## 

Small-volume hypertonic fluid resuscitation has been investigated extensively, especially in hemorrhagic shock [1]. The immediate effects include intravascular volume expansion, restoration of cardiac output and blood pressure, and possibly improvement of regional and microcirculatory blood flow. Hypertonic resuscitation also exerts immunologic and anti-inflammatory effects, which may be of potential benefit in the early resuscitation and management of septic shock [2]. Different conventional hemodynamic optimization strategies in septic patients result in distinct biomarker patterns [3]. In experimental human endotoxemia, prehydration shifts the cytokine pattern toward a more anti-inflammatory state and results in less clinical sepsis symptoms, suggesting an association between the inflammatory response and the hydration or resuscitation status of septic patients [4]. In addition, but mainly based on preclinical studies, volume resuscitation with hypertonic fluids may exert intrinsic beneficial effects by modulating the inflammatory response and apoptosis in trauma and sepsis; these effects however have not yet been convincingly shown in clinical studies [5,6]. In healthy volunteers, hypertonic fluid administration results in attenuation of neutrophil cytotoxicity and inhibition of the interaction between neutrophils, platelets, and endothelium [7]. Hypertonic saline alters neutrophil cell shape, resulting in cytoskeleton remodelling, which has implications for signal transduction and the cytotoxic response. The anti-inflammatory effects on neutrophils, oxidative burst, and cytokine release are mediated through the signalling molecule mitogen-activated protein kinase (MAPK) p38 and suggest the existence of an osmolarity sensing system in immune cells of humans [8,9].

The immune response during sepsis is complex and involves a network of control elements that includes pathogen-associated molecular patterns, cell adhesion molecules, pro- and anti-inflammatory mediators released by activated macrophages, and complement activation. Plasma levels of inflammatory mediators in sepsis reflect the overflow of these mediators into the bloodstream and may give limited insight into the actual activation of the leucocytes and the innate immune system [10]. In a previous study, we have described the use of real-time reverse transcriptase polymerase chain reaction (RT rtPCR) to quantify inflammatory mediator expression in circulating leukocytes of septic patients [11].

This study was designed to quantify the changes in inflammatory mediator gene expression in circulating leukocytes, obtained from septic shock patients who were randomly assigned to receive a bolus of hypertonic or isotonic fluid.

## Methods

Following approval by the Northern Y Regional Ethics Committee (NTY/06/08/070), we conducted a single-center, double-blind prospective, randomized, controlled study in the Intensive Care Unit of a tertiary referral teaching hospital. Informed consent was obtained from patients or their nearest relative. This study is part of a trial that investigated the cardiovascular effects and the effects on gastric and sublingual microcirculation of hypertonic and isotonic resuscitation, which will be published separately. The trial is registered with ANZCTR.org.au as ACTRN12607000259448.

### Study protocol

Consecutive adult patients with septic shock were screened for inclusion in the study. Septic shock was defined according to standardized criteria [12]. Patients were randomized to receive intravenous administration of 250 ml of NaCl 7.2%/6% hydroxyethylstarch (hypertonic group, HT) or 500 ml of 6% HES (isotonic group, IT) over 15 minutes. Hemodynamic measurements, echocardiography, tonometry, and SDF imaging of the sublingual microcirculatory blood flow will be described in a separate paper. Blood samples were taken from the arterial catheter at baseline and after 4, 8, 12, and 24 hours after fluid infusion for further analyses.

### Laboratory methods

Real-time reverse transcriptase polymerase chain reaction (RT rtPCR) was used to quantify mRNA expression of different sepsis mediators in peripheral leukocytes. Based on their importance in the immune response and pathology of sepsis, we chose ten representative genes from a variety of different groups of sepsis mediators: inflammatory cytokine interleukin-6 (IL-6), anti-inflammatory cytokine interleukin-10 (IL-10), chemokine interleukin-8 (IL-8), intercellular adhesion molecule-1 (ICAM-1), monocyte chemoattractive protein-1 (MCP-1), tissue factor (TF), integrin cluster of differentiation molecule CD11b, L-selectin, and matrix metalloproteinase-9 (MMP9). To standardize and normalize the amount of biological material between samples, a suitable housekeeper gene (β2 microglobulin, B2M) was chosen [13,14]. Table 1 shows the abbreviation, major activity, and the source of expression of the investigated mRNA transcripts. A housekeeper gene was used to correct for the absolute amounts of total mRNA variations between different samples. All primers (Sigma, Australia) were optimized for use by amplification of cDNA using reverse transcriptase PCR and the resulting amplicons sequenced for confirmation. To quantify the level of hypertonicity that was achieved, plasma sodium levels [Na^+^] were measured every 30 min using a point-of-care blood gas analyzer (ABL 800 Flex, Radiometer, Copenhagen). To compare the magnitude of plasma volume expansion, dilution of hemoglobin (Hb) concentration was assessed 1 hour after the fluid administration.

**Table 1:** Sources and biological effect of investigated inflammatory mediators

Inflammatory mediator	Abbreviation	Major cell sources	Major activity
Interleukin 6	IL-6	T cells, macrophages	Mediator of fever and acute phase response. Has both pro- and anti-inflammatory properties

Interleukin 8	IL-8	Macrophages, epithelium, endothelium	Mediator inflammatory response. Chemotactic mainly for neutrophils

Interleukin 10	IL-10	Monocytes, lymphocytes	Anti-inflammatory, inhibits synthesis various pro-inflammatory cytokines

Intercellular adhesion molecule 1	ICAM-1	Leucocytes, endothelium	Facilitates leucocyte endothelial transmigration, signal transduction pro-inflammatory pathways

Monocyte chemoattractant protein 1	MCP-1	Monocytes, endothelium, smooth muscle cells	Chemotactic mainly for monocytes

Tissue factor	TF	Subendothelial tissue, platelets, leucocytes	Initiation coagulation cascade, intracellular signalling (angiogenesis, apoptosis)

Cluster of differentiation molecule 11b	CD11b	Monocytes, neutrophils, macrophages, natural killer cells	Regulates leucocyte adhesion and migration, implicated in phagocytosis and cell mediated cytotoxicity

L-selectin	L-selectin	Leucocytes	Adhesion and homing receptor for leucocytes to enter secondary lymphoid tissues

Matrix metalloproteinase 9	MMP9	Macrophages, neutrophils, endothelium	Breakdown extracellular matrix, invasion of inflammatory cells

β2 microglobulin			Housekeeping gene

### Laboratory protocol

One milliliter of blood was added to 4 ml of 5 M guanidine thiocyanate (GITC) solution to preserve the RNA. Total cellular RNA was isolated from the cell samples using the following method: 0.5 ml 2 M sodium acetate (pH 4), and 2.0 ml of 100% ethanol were added to the GITC-lysed blood and the sample mixed and allowed to stand in an ice bucket for 10 min before being centrifuged for 15 min at 15,000 (g) at 4°C. The supernatant was carefully decanted so as not to disturb the pellet, which was resuspended in 0.5 ml of GITC solution and then mixed. When the pellet was dissolved, 50 μl of 2 M sodium acetate was added followed by 0.5 ml of water-saturated phenol. The solution was placed on ice for 10 min and then 200 μl of chloroform added and the tube vortexed before being centrifuged at 16,000 g for 10 min. The top layer was removed to new tube and an equal volume of 100% Analar Isopropanol added and mixed by invertion, following which the sample was placed on ice for 10-15 min to precipitate the total RNA. The tube was recentrifuged at 16,000 g for 10 min, the supernatant removed and the pellet resuspended in 1 ml of 70% ethanol, and then centrifuged at 16,000 (rpm or g) for 5 min. The ethanol was removed and the pellet briefly air dried. The pellet was resuspended in 18 μl of Tris/Mn RNA buffer and 2 μl of Promega DNase solution was added and the sample incubated, shaking at 37°C for 30 min to digest any contaminating DNA. A total of 2 μl of Stop solution was added, heated, and shaken at 65°C for 10 minutes, with the samples then put on ice. Quality and quantity of RNA checked on a Nanodrop instrument by measuring absorbance at 260-, 280-, and 203-nm wavelength.

A cDNA copy of total RNA was prepared using the SuperScript III reverse transcriptase first strand cDNA synthesis kit (Invitrogen, Carlsbad, CA) according to the manufacturers instructions, using oligo(dT)15 (Roche Molecular Systems, Pleasanton, CA) to prime the reactions. Briefly, reverse transcription reactions were performed using a PTC200 DNA engine (BioRad, Hercules, USA) in tubes using 1.0 to 1.5 μg RNA, 1 μL of 50 μM Oligo dT (Roche, Auckland, NZ), and sterile MQ water to achieve the desired volume. The tube was then heated to 70°C for 5 min to destroy any RNA secondary structures. The tubes were cooled on ice before the reverse transcriptase components were added. The enzyme mix for each sample contained 2.5 μL of sterile MQ water, 4 μL 5X first strand buffer, 1 μL of 0.1 M DTT, 1 μL of dNTP mix (10 mM), to which was added 0.5 μL of SuperScriptIII (Invitrogen). This was added to the 0.2-mL tubes containing the RNA and Oligo dT mixture using an electronic dispenser, mixed, then spun down (5 k rpm for 20 seconds) and left at 25°C for 5 minutes before incubating at 50°C for 1 hour. The reaction was then halted by heating at 70°C for 15 minutes.

A check of cDNA production was performed by amplification of the housekeeping gene β_2 _M using 0.5 μL cDNA samples with negative controls. The cycle time and temperature settings were initially 95°C, 2 minutes; then 40 repeating cycles of 94°C, 20 seconds; 55°C, 20 seconds, 68°C, 30 seconds; before a final step of 68°C for 5 minutes. The cDNA samples were stored at -70°C until used in reverse transcriptase polymerase chain reaction (rtPCR).

### Real-time rtPCR quantification

PCR products were labelled with SYBR^® ^82 (Invitrogen). RT rtPCR was performed in 100-μL thin-walled tubes (Corbett Research) and monitored in a Rotor-Gene™ 6000 (Corbett Research). Each 20-μL reaction mixture contained real-time PCR Mastermix (10× Thermostart^® ^Reaction Buffer (AB Ltd.), 1/20,000 dilution of SYBR^® ^82, 5 mM MgCl_2 _pH 8.5, 0.5 U of ABGene Thermostart^® ^DNA polymerase (AB Ltd.), and 5 pmol of forward and reverse primers), and approximately 1 μL of cDNA.

Following an initial denaturation step at 95°C for 15 minutes, 40 cycles were performed using 94°C for 20 seconds, annealing at 55°C for 20 seconds, extension at 68°C for 30 seconds, and fluorescence acquisition at 80°C for 10 seconds using the yellow channel (excitation at 530 nm, detection at 555 nm).

Following amplification in each run, a dissociation melt curve was determined. PCR products were heated from 75°C to 99°C in 0.5°C increments every 5 seconds. All melt curves showed a single peak consistent with the presence of a single amplicon. Each reaction was run in duplicate, and the Ct values (Roto-Gene software, version 1.7) and PCR efficiencies were averaged [15]. The mean Ct and PCR efficiency values were used to estimate the initial copy number (ICN) of mRNA transcripts of each particular gene, including the house-keeping gene (B2M) [16]. To correct for different concentrations of mRNA, 1 μg of total RNA was used to make cDNA and then the ratio of the Housekeeper gene to the gene of interest was used. Because the housekeeper gene is not affected by the treatment, the Ct and the efficiency of amplification could be used to adjust for significant difference in the starting concentration of mRNAs. Specimens in which the RNA yield, quality, or amplification efficiency were compromised were rejected for analysis.

### Data analysis

The level of gene expression was quantified using the initial copy number. We did a logarithmic transformation on this number to achieve a normal distribution of the data and hence to allow the use of repeated measures analysis of variance (ANOVA). "Treatment-group" was the between-subject variable, and "time" was the within-subject variable. The "time×treatment-group" interaction term was the indication of the evolution of different responses between the two treatment groups. We used the Tukey-Kramer Multiple-Comparison test for post-hoc comparisons at different times. The Student test was used to compare parameters with a normal distribution, and the effects on nonnormally distributed parameters were compared by using the Mann-Whitney test and the Wilcoxon signed-rank test for paired measurements. Bonferroni correction was used to adjust for multiple (n = 7) comparisons. Using this correction, *p *< 0.0071 was considered to be significant. All statistical calculations were performed using NCSS 2007 (version 07.1.13, NCCS, Kaysville, UT).

## Results

Baseline characteristics are shown in Table 2. The treatment groups had similar severity of disease, as expressed by APACHE II and SOFA scores. All patients required vasoactive drugs for hemodynamic support as required for the diagnosis of septic shock. None of the patients received immunosuppressive agents, such as steroids before or during the study. No differences in baseline counts of white blood cells (WBC) and polymorphonuclear cells (PMN) were present (Table 2).

**Table 2:** Baseline characteristics

Variables	IT group (n = 12)	HT group (n = 12)	*P *value
Age (yr)	61 ± 13	56 ± 16	0.45

Men	6 (50%)	7 (58%)	0.68

APACHE II	23.5 ± 7.4	24.4 ± 6.7	0.75

SOFA	8.9 ± 2.5	9.8 ± 3.4	0.5

WBC (×10^9^/l)	10.7 [7.4-14.5]	14.9 [6.7-35.6]	0.3

PMN (×10^9^/l)	9.7 [6.4-12.9]	13.1 [9.9-28.3]	0.28

Source of sepsis			

Abdominal (n = 10)	5	5	

Pneumonia (n = 8)	5	3	

Soft tissue (n = 3)	1	2	

Other (n = 3)	1	2	

APACHE, Acute Physiology and Chronic Health Evaluation; SOFA, Sequential Organ Failure Assessment; WBC, white blood cell count; PMN, polymorphonuclear leucocytes.

Data are presented as mean ± SD, as numbers (%) or as median [interquartile range].

### Gene expression at baseline

**Figure 1:**
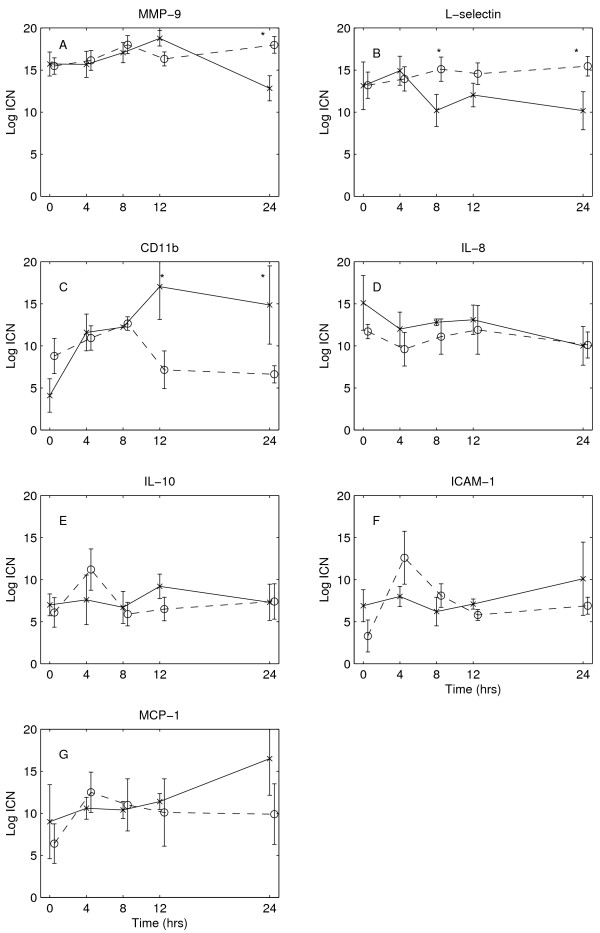
**Changes in inflammatory mediator genes over time for the two treatment groups**. Hypertonic group, *solid line*; isotonic group, *dotted line*. Data are expressed as mean (SD) of the logarithm of the initial copy number.

The expression at baseline of all measured mediators was comparable between the two groups (Figure 1). The genes IL-6 and TF were insufficiently expressed to use for further data analysis. Patients with abdominal sepsis had significantly more variability in the baseline gene expression compared with the other sepsis patients (SD 4.1 ± 0.8 vs. 2.8 ± 0.8, *p *= 0.03). The expression of MMP9 in patients with abdominal sepsis tended to be higher compared with patients with pulmonary or other sepsis (16.5 ± 3.5 vs. 13.9 ± 2.6 and 15.2 ± 3.7), but this difference did not reach statistical significance (*p *= 0.21 and *p *= 0.57, respectively).

### Treatment effects

In the HT group, [Na+] increased from 135 ± 5 mmol/l at baseline to 143 ± 7 mmol/l after 30 min (*p *< 0.0001) and decreased to 140 mmol/l after 2 hours and did not change after that. This increase corresponds with a plasma osmolality of approximately 300 mOsm/kg. No significant change in [Na+] was found in the IT group. The Hb concentration before the fluid infusion was not statistically different between the groups (HT 108 ± 15 g/l, IT 96 ± 17 g/l; *p *= 0.07). In both groups, fluid administration significantly decreased Hb after 1 hour (HT 99 ± 15 g/l, *p *< 0.00001; IT 84 ± 15, *p *< 0.00001). The magnitude of hemodilution as assessed by the difference in Hb after 1 hour was similar between groups (HT 9.0 ± 2.2 g/l, IT 11.6 ± 4.5 g/l; *p *= 0.09). The WBC count following study fluid administration did not change significantly from baseline (IT 11 [8-17] × 10^9^/l, *p *= 0.54; HT 17 [11-25] × 10^9^/l, *p *= 0.52) and was not different between the treatment groups (*p *= 0.15). The PMN count after treatment also was not different from baseline (IT 10 [7-16] × 10^9^/l, *p *= 0.44; HT 15 [8-22] × 10^9^/l, *p *= 0.98) or between groups (*p *= 0.28).

The expression of the investigated genes over time in both treatment groups is shown in Figure 1. MMP9 showed a significant effect over time (ANOVA, *p *= 0.001, expression at 24 hr different from expression at 8 hr and 12 hr (post-hoc test)) and the interaction term (ANOVA, *p *= 0.0002). This indicates that the MMP9 expression at 24 hr decreased in the HT group, whereas in the IT group the MMP9 expression was still elevated (Figure 1A). L-selectin expression also was more suppressed after more than 4 hr in the HT group compared with the IT group (ANOVA, *p *= 0.007; Figure 1B). CD11b showed a nonsignificant increase in expression over the first 8 hr (time ANOVA, *p *= 0.04), an effect that was more pronounced in the HT compared with the IT group (ANOVA, *p *= 0.02). However, after 12 hr, the levels returned to time = 0 levels in the IT group, but remained elevated in the HT group (Figure 1C). The other mediators ICAM, IL8, IL-10, and MCP-1 did not show any significant changes over time or between treatment groups (Figure 1).

## Discussion

In this study, we examined the effects of hypertonic versus isotonic fluid administration on circulating leukocyte expression of important sepsis mediators in septic shock patients. To our knowledge, this has not been studied before in this group of patients.

Hypertonic fluid administration resulted in a different gene expression pattern compared with isotonic fluid. In the HT group, the expression of MMP9 and L-selectin was suppressed compared with the IT group. CD11b tended to remain elevated after 12 hr in the HT group while returning to baseline in the IT group.

Our study has several limitations. Septic shock patients are not a homogenous population, and the expression of inflammatory mediators is highly variable and not only dependent on the source of sepsis but also on the genetic make up of the host, which defines the immune response [17]. We did not directly measure inflammatory mediator peptide levels in the peripheral blood, which is the more common way to study the immune response to sepsis. The levels and dynamics of these mediators correlate with outcome [18-20]. One of the main problems when measuring inflammatory mediator peptide levels in the peripheral blood is that only the endocrine overflow is measured, not the local autocrine and paracrine receptor binding effects [10,11]. On the other hand, measuring expression of the inflammatory mediator genes may not reflect the functional activity of the end-protein, because this also depends on translation and various posttranslational modifications that determine whether the protein becomes active. Currently there are no methods to measure functional protein activity reliably. In addition, inflammatory gene activation tends to be a slow process and can take many hours depending on the gene measured. This is in contrast to the immediate and short-term changes observed in inflammatory mediator peptide levels in peripheral blood and could account for the time course of changes found in our study. In addition, our methodology does not allow us to distinguish between direct effects and indirect effects, e.g., downstream in a cascade of events, or induced by a change in the level of inhibition. Also, our measurements were limited to circulating leucocytes, and thus our study does not provide information on gene expression in adherent or migrated neutrophils. Even cell separation procedures would not be able to detect inflammatory mediator expression in cells within the tissues. Furthermore, the level of hypertonicity that was achieved in the HT group may not have been optimal to significantly influence immune function. It has been proposed that the level of hypertonicity should probably exceed 330 mOsm/kg to benefit patients in terms of immune function [21]. Finally, hydroxyethyl starch solutions have been shown to have an effect on markers of inflammation and endothelial injury [22]. The two patient groups in our study received a different amount of hydroxyethyl starch, which theoretically could exert a different effect on the gene expression of inflammatory mediators, although this effect may not be dose-dependent.

MMP9 is released from granules of neutrophils and induces capillary leakage by degrading endothelial membranes. High plasma levels of this inflammatory marker as well as high mRNA expression in septic patients have been reported previously [11,23,24]. Both plasma MMP9 concentrations and monocyte MMP9 mRNA levels were significantly higher in nonsurvivors than in survivors of septic shock [24]. Hypertonic fluid administration has been shown to reduce capillary leakage and improve capillary blood flow in several studies [6,25]. This effect has been attributed mainly to the direct osmotic effects on endothelial cell swelling and luminal narrowing [26,27]. Our finding of suppression of MMP9 could be used to generate an alternative hypothesis by which hypertonic fluids may reduce capillary leakage and edema formation, which should be investigated further. Although we did not specifically investigate the degree of capillary leakage in our study, we did find that patients treated with hypertonic fluid needed significantly less fluid in the following 24 hours compared with patients in the IT arm (HT 2.8 ± 1.5 liter/24 hours vs. IT 4.1 ± 1.6 liter/24 hours, *p *= 0.046).

L-selectin is a transmembrane glycoprotein expressed on leucocytes, involved in rolling and adhesion of leucocytes along vessel walls adjacent to the site of injury. The binding through L-selectin is dependent on sufficient shear stress above a critical threshold, to promote and maintain rolling interactions [28]. In our study, expression of L-selectin was depressed in the HT group. This finding is consistent with previous findings and suggests that hypertonic fluid modulates the immune response by preventing neutrophil adhesion to the endothelium [2,29-31]. In several animal models of shock, intravital microscopy was used to visualize neutrophil rolling and adhesion to the endothelium in a real-time fashion. Hypertonic resuscitation has been shown to decrease neutrophil rolling and adherence [6,25].

The mediator CD11b is member of the integrin family, which is responsible for adhesion of leucocytes to endothelial cells. These integrins are expressed constitutively and kept largely in an inactive state to undergo in situ activation upon leukocyte-endothelial contact by both biochemical and mechanical signals. This activation process takes place within fractions of seconds by in situ signals transduced to the rolling leukocyte as it encounters specialised endothelial-displayed chemoattractants [32,33]. Our finding of a possible trend toward elevated gene expression of CD11b after 12 hours in the HT group compared with control is not easy to interpret. Rizoli and coworkers showed in animal models of hemorrhagic shock that hypertonic fluid prevents LPS-stimulated expression and activation of CD11b in the lung [34,35]. In a randomized, controlled study by the same group in patients with traumatic hemorrhagic shock, hypertonic fluid abolished shock-induced CD11b up-regulation [36]. There are important differences between these studies and ours that could account for the different findings. Hemorrhagic shock and septic shock are distinctly different disease processes with important differences in immune response. Furthermore, timing of the intervention may be important [37]. In the animal experiments described, hypertonic fluid was given before the LPS challenge, which is obviously unachievable in patients already in septic shock.

We were unable to measure sufficient expression of the inflammatory genes for IL-6 and TF to include them in our analysis. During sepsis, the vast majority of circulating leucocytes are neutrophils, with hardly any circulating monocytes, because these are known to migrate out of the circulation. This means that our measurements essentially targeted gene expression in neutrophils, whereas IL-6 is mainly expressed in monocytes and TF in (sub)endothelium. In other words, despite high plasma protein levels of IL-6 in sepsis, the actual gene expression in circulating leucocytes is expected to be very low. Another or contributory explanation could be that high blood levels of inflammatory peptides may result in homeostatic suppression of the associated genes.

Similar to our previous study [11], there was a trend toward increased expression of MMP9 in patients with abdominal sepsis compared with other forms of sepsis, although in the present study this difference did not reach statistical significance. This observation reiterates that the inflammatory response in sepsis is heterogeneous depending on the source and the infecting organism.

In conclusion, we have shown that in septic shock patients, hypertonic fluid, compared with isotonic fluid, may modulate expression of several, but not all, measured genes that are implicated in neutrophil-endothelial interaction and capillary leakage. To our knowledge, this is the first study to report the effects of hypertonic resuscitation on inflammatory gene expression in septic shock patients.

## Competing interests

The authors declare that they have no competing interests.

## Authors' contributions

FvH designed and conducted the clinical study, and drafted the manuscript. JS participated in the design of the study and performed the statistical analysis. RC designed and carried out the laboratory measurements. MLP is the research coordinator responsible for the clinical trial and the data collection. PP and JvdH conceived of the study and contributed to the manuscript. All authors read and approved the final manuscript.

## Disclosures

The study was supported by a grant from the Waikato Medical Research Foundation (WMRF 127). Dr. van Haren has no conflicts of interest to disclose. Dr. Pickkers has no conflicts of interest to disclose. Mr. Cursons has no conflicts of interest to disclose. Prof. Sleigh has no conflicts of interest to disclose. Mary La Pine has no conflicts of interest to disclose. Prof. van der Hoeven has no conflicts of interest to disclose.
